# Defining Self-Management for Solid Organ Transplantation Recipients: A Mixed Method Study

**DOI:** 10.3390/nursrep14020073

**Published:** 2024-04-17

**Authors:** Katie Brunner, Lydia Weisschuh, Stefan Jobst, Christiane Kugler, Anne Rebafka

**Affiliations:** Institute of Nursing Science, University Medical Centre, Albert-Ludwigs University Freiburg, Breisacher Straße 153, 79110 Freiburg, Germanystefan.jobst@uniklinik-freiburg.de (S.J.); christiane.kugler@uniklinik-freiburg.de (C.K.)

**Keywords:** self-management, Solid Organ Transplantation, scoping review, conceptual definition, content analysis

## Abstract

Patients with Solid Organ Transplantations (SOTx) face long-term lifestyle adaptations, psychological and social adjustments, and complex self-care regimes to maintain health post-transplant. Self-management (SM) skills represent important aspects of nursing communication with SOTx patients; however, there is potential for SM to be defined narrowly in terms of medication adherence. The study presented here collated the existing definitions in a mixed method review in order to identify SM attributes for this group (including those unique to this population). Secondary analysis of a dataset and bibliographic analysis and an expert panel were used to develop a comprehensive working definition of SOTx patients. The analysis comprised critical interpretation of the evolving definition content, concepts, and contexts of application in current usages and over time. We identified eight definitions and 63 cited definition sources from bibliographic analysis. Findings identified limitations of the existing definitions. Population-specific attributes included optimisation of transplant outcomes, active engagement in healthy behaviours, control, structure, and discipline characteristics, and moderating factors of patient motivation, self-efficacy, and cognitive function. A critical appraisal of definitions indicated inadequately defined aspects such as setting, temporal dimension, concept interaction, interventions, and measurable outcomes. The bibliographic analysis highlighted the influence of broader chronic illness constructions of SM, underpinning the generalisable SM attributes in current definitions. Further research may advance the development of a definition in exploring the relevance of SOTx-specific attributes of the definition.

## 1. Introduction

### 1.1. Background

#### 1.1.1. The Approach of This Study

Solid Organ Transplantation (SOTx) recipients represent one group of many of patients who manage their own care in partnership with health care providers—a concept referred to as self-management (SM). The unique needs of SOTx patients have yet to be integrated into a set of attributes within the SM framework. This article will explore and define SM attributes in relation to SOTx patients, presenting definitions currently in use and a bibliographic analysis of their evolving application. This paper also presents a working definition. In presenting this mixed method study, we underline here principles that informed our approach. Our broader programme of research required a definition specific to SOTx patients for subsequent patient intervention. Therefore, this study sought to define SM from within the empirical study literature base associated with our population of interest. This study interprets the concept of SM predominantly at the level of attributes associated with the conceptual construct. Our approach considered the possibility that the definitions identified may refer to general SM definitions, contain SOTx-specific definitions, or a combination of the two. A key principle was that the analysis would not focus purely on the unique defining elements for SM in SOTx patients as opposed to more generalisable attributes of SM. Given the variation in conceptualising this field, our study stages set out to understand the existing definitions in context and to appraise the appropriateness of definition attributes, hence a systematic approach is used. The design of this mixed method study, therefore, goes beyond identification and compendium of current definitions. Bibliographic analysis provided critical insight into the application of definitions over time (identifying unique SOTx-specific or general SM attributes) in order to contextualise and critically judge the comprehensive group of attributes to be included in any revised working definition.

#### 1.1.2. Self-Management and SM Attributes

The concept of self-management can be traced back in the UK to the start of the 20th century, with care costs mounting and the policy agenda increasingly seeking health care and health service delivery solutions that are more patient-focused and recognise the central role of patients in the care process [1]. A recent delineation of self-management support types includes the following: information about condition and/or its management, provision of/agreement on specific clinical action plans, regular clinical review, monitoring of condition with feedback, practical support with adherence—medication or behavioural—provision of equipment, training/rehearsal for everyday activities, training/rehearsal for practical self-management activities, training/rehearsal for psychological strategies, social support, and lifestyle advice and support [2]. Thus, health professional support acknowledges the changing patient role and the expectation of increased active involvement in care through SM [3].

#### 1.1.3. SOTx Patients and Rationale for Augmented Definition of SM

Globally, there were 129,681 solid organs transplanted during 2020, the most common being kidney and liver transplants [4]. Transplantations become necessary when the recipient’s organ has failed due to disease or injury. Beneficial outcomes of transplantation are increased survival rates and improvements in quality of life [5]. Worldwide, principles of successful transplantation extend to post-transplant health in long-term follow up [6].

Following SOTx transplant, possible risks that require patient management include organ rejection or infections leading to graft failure or loss, a dimension absent in chronically ill populations who self-manage. For SOTx patients, absence or insufficient attention to self-management can result in such complications [4]. Typically, patients need to monitor signs of infection, medication side-effects, and physical and psychological status and implement healthy lifestyle changes (such as a special diet, psychosocial problems, mental health problems, and substance use) [5,6,7,8,9,10,11]. In terms of a medication regimen, patients must learn how to calculate dosages of interacting medications. It is this complexity of monitoring and medicating which constitutes another dimension of difference between self-management of this and other groups. Monitoring also includes looking out for a broad spectrum of common conditions, contraception and pregnancy planning in women, vaccinations, travel planning and precautions, and screening for conditions such as osteoporosis are preventative considerations common to all transplant recipients [12]. As these additional patient needs for the management of symptoms began to be recognised, SM and chronic illness frameworks were adopted [13].

In clinical practice, evidence suggests that nurses experience difficulties operationalising self-management support for SOTx patients [14]. As Been-Dahmen points out, multiple perceptions exist about how to define and operationalise SOTx forms of SM support, and these can be interpreted narrowly due to clinically critical management types such as measures that seek to assist patients in following a strict regimen of immunosuppressive medication for maintaining graft functioning and the difficulties experienced with adherence. Therefore, the post-transplant period has tended to focus on promoting medication adherence and self-monitoring over psychological and social demands [14].

Service provision for supporting self-management could be developed further to include patient education programmes [7,15] to enable patients to acquire skills related to medical management and to educate about how to live a full life with illness [15]. Across European contexts, interventions have been developed to evidence the utility of post-transplant patient education (some combining increasing informational needs with SM skills) [7,16,17], despite the evidence that increased graft survival may be a result of improved immunosuppression, management of comorbidities, and education of patients [18]. Nurses have a key role in interacting with, educating, and supporting this group of patients to maximise independence in patients initiating their own care ([18]). Although, the presence of a specialised nurse is not standardised in European countries, such as Germany [19]. This means that the range of defining attributes for patient SM is likely to be variable and potentially under-supported. There are examples of conceptual analyses of SM, however, these are not focused on SOTx populations. Johnston et al. [1] published a conceptual review of SM in the context of palliative nursing. The authors found that SM support (e.g., helping patients to maintain normality, independence, and control) could provide an increased level of patient and family-centred care. Thus, findings from this in-depth approach help to elucidate key attributes and the SM aspects which can contribute to forms of health care delivery such as more personalised care.

#### 1.1.4. A Brief Historical Context for SM and Chronic Illness Research

In undertaking a study of definitions of SM, including a bibliographic analysis, it is necessary to briefly set the scene for the historical origins of SM in chronic illness research. The foundations of the concept of SM lie in the seminal research by Corbin and Strauss on chronic illnesses [20,21,22]. As such, syntheses can be identified in this broader field, and there are several examples of narrative reviews that seek to collate SM intervention findings for long-term conditions (multi-morbidities [23], arthritis [24,25], and chronic conditions [26,27,28]). Audulv [29] notes that the emergence of SM as a term occurred in the 1960s and 1970s in the context of shifts towards self-help frameworks, a power imbalance in the traditional doctor–patient relationship, and the medicalisation of former non-medical aspects of life [30]. The theory in this field addresses some of the problems of managing chronic illness at home. Management consist of the concept of “work” in the areas of (1) illness work, (2) everyday life work, and (3) biographical work [20]. This conceptualisation of tasks was highly influential in further theorisation [24]. Been-Dahmen remarks that conceptualisations of SM shifted from assessing improvement in clinical outcomes, often medication adherence (reductive in nature), to broader social and psychological conceptualisations (such as Lorig and Holman’s [25] SM tasks [3]). In the context of chronic care, Lawn et al. suggest the conceptualisation of concordance for SM whereby “‘[frank information, negotiation and a spirit of cooperation]’ is expected” [27] (p. 206, Table 1). This is in contrast to compliance medication adherence conceptualisations that reflected “active engagement in and ownership of a behavioural change process towards improved health by the person with the health condition” (op cit). Therefore, definitions emerged encompassing the individual’s ability to manage the symptoms, treatment, physical and psychosocial consequences, and lifestyle changes through the patient’s ability with cognitive, behavioural, and emotional responses [31].

### 1.2. Study Rationale

A recent review demonstrated the continuing need to assess the range of complex physiological, psychological, social, and spiritual issues that arise for patients in the process of organ transplantation, requiring close assistance from medical teams [32].

To date, no generally accepted definition of SM for SOTx patients exists, despite growing interest in SM for this group, nor has there been a synthesis in this field. Abtahi et al. [7] recently reviewed SOTx SM interventions and pragmatic solutions to enhance self-management skills, defined through patient tasks and abilities to manage symptoms with family and healthcare professionals using patient-centred care approaches for organ transplantations. However, the authors did not analyse varying definitions of SM across sources. Therefore, the adequacy or appropriateness of the existing definitions for SOTx groups is particularly important given the context of chronic illness SM constructions and the potential for definition constructions to omit unique SOTx aspects or to be overly narrow in focusing on medication adherence. Richard and Shea [33] suggest conceptual clarity can provide stable patterns of concept utilisation, increased precision in the identification and measurement of concepts in research questions, and greater precision in healthcare delivery and intervention goals. Other authors have also advocated for more clearly defined terms such as SM self-care [34] and social support [35]. Therefore, definitions of SM are a resource for nurses and other professionals (especially in the absence of professional guidance for SM [36]).

This study is a part of a larger research project called the SMART study [36], consisting of a synthesis of evidence on aspects of SM after SOTx in preparation for intervention development. This scoping review focused on international empirical literature on self-management, self-management support, or recipients’ or healthcare providers’ perspectives of challenges and needs potentially addressable by SM.

### 1.3. Study Objectives and Research Questions

Study objectives were (1) to identify and critically review the existing definitions for SOTx SM in empirical research and (2) to propose a comprehensive definition of SM for SOTx informed by feedback from an expert panel—by asking the following research questions: how has the concept of SM been defined in the literature for the patient group SOTx [36]? What are the characteristics and conceptual underpinnings of these definitions, and are they considered adequate?

## 2. Materials and Methods

### 2.1. Identification of an Approach

As previously stated, this study set out to produce a definition for SOTx populations based on existing definitions and a bibliographic analysis to provide critical insight into the application of definitions over time. Three sources that have explored definitions guided our methodological approach: Sørensen et al. [37] (a content analysis), Johnston et al. [1] (concept analysis), and Williams et al. [35] (critical appraisal). By combining elements of these approaches, we aimed to critically review definitions and related concepts [34]. The definition produced by this study was intended to be stipulative [38], that is, terms providing defining attributes or characteristics of a phenomenon that set out rules for the way in which it is constructed and how conceptual components may fit together.

This section summarises the central tenets of the three methodological approaches that we engaged with. First is content analysis of definitions developed by Sørensen et al. [37]. The defining features of this approach included the categorisation of the definitions’ components into so-called clusters of meaning and the discussion of the findings with a panel of experts to create a comprehensive “all-inclusive” definition. The authors describe the process as an examination of clusters, discussed and condensed by the research team, capturing the essence of the definitions.

**Figure 1:**
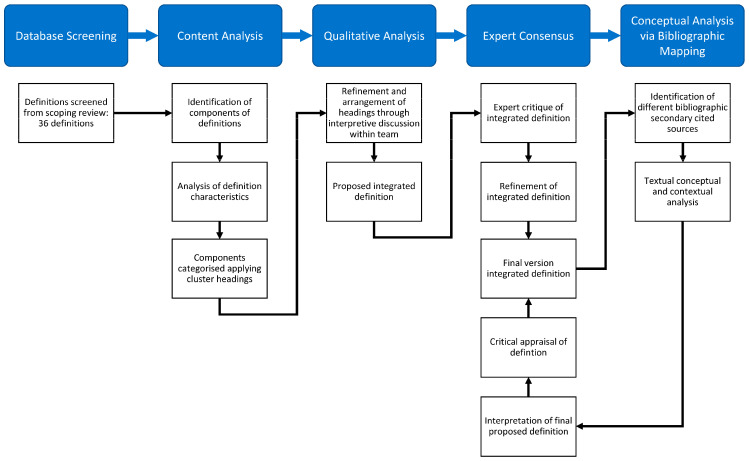
Study design [36].

Secondly, Johnston et al.’s [19] conceptual analysis of definitions (as opposed to a conventional concept analysis technique) was “to clarify meanings and develop operational definitions, through considering evidence from multiple disciplines and sources” (p. 2). This approach is a modified version of Walker and Avant’s nursing-based strategy for theory construction (2010) [38], involving conceptualisation of concepts to the nursing practice to provide standardisation of the nursing language. In addition, the concepts categorise information into meaningful constructs when applied to a phenomenon. The methodology requires the identification of conceptual attributes, contributing factors which must be present before the occurrence of the concept (antecedents) and events that occur as a consequence of the concept (consequence) to understand contexts of the application of the definition. The third methodology which informed this study was by Williams et al. [35], who provided an approach to judging the appropriateness of definitions through (1) assessment of attributes and (2) making judgements about the maturity of the concept and its appropriateness. Individual methods consisted of the identification of definitions within the SMART study scoping review records’ database, content analysis to identify definition components (involving collaborative research team qualitative analytical approaches to configure conceptual components into a new definition), a definition concept analysis (via bibliographic analysis and the collation of a secondary pool of definition sources), and an expert consensus exercise. Figure 1 displays the methodological design. Prior to secondary bibliographic searching, the definitions to be analysed in our study were taken from the SMART study screened database.

### 2.2. Definitions Currently in Use—Study Identification via Secondary Analysis of SMART Scoping Study Dataset

This section now describes our methodology as a process. In the absence of reporting guidelines tailored to definition and bibliographic analysis (including critical interpretive elements), this paper referred to the RAMESES guidelines [39] for reporting a meta-narrative review. This reporting guideline is aimed at reviews seeking to illuminate a heterogeneous topic area by highlighting the contrasting and complementary ways researchers have studied the same or a similar topic (Supplementary File S1). There were no changes to the review questions for the identification and eligibility of definitions during the review. The findings for the broader SMART study scoping review, of which this is a sub-study, will be reported separately.

We began with the identification of existing definitions. In brief, the initial set of definitions from the SMART study scoping review conducted from inception to September 2021 was obtained. A protocol detailing the SMART study search design has been published elsewhere [36]. The SMART study scoping review included 742 records (initially 34.045 records from database and supplementary searches). These 742 comprised the dataset for this definition-focused sub-study prior to secondary source searching. Six electronic databases, three study registers, and the Deutsches Register Klinischer Studien were searched, supplemented by hand-searches, reference checking, and expert recommendations. Terms included “SOTx”, “self-management”, and the “perspective of recipients/HCP”. Types of sources were restricted to journal articles (primary studies, evidence syntheses), published protocols, and conference papers.

### 2.3. Definitions Currently in Use–Selection of Sources of Evidence and Eligibility Criteria

This study applied a second screen to the definitions identified from the included 742 SMART study records containing a definition of SM for SOTx. The broader SMART study eligibility criteria focused on published articles on self-management of challenges and needs, published in English or German reporting on adults after SOTx, that is, heart, lung, liver, pancreas, kidney, or small bowel transplantation; it is reported in full elsewhere [36]. We aimed to identify definitions of self-management (including the term self-management support) but not associated concepts such as self-efficacy or self-care.

Papers were eligible if they contained a definition consisting of the following: an explicit definition or an extract defining SM—reviewers made a judgement if the extract was fulfilling the function of a definition. The source type was either journal articles (primary studies, evidence syntheses), published protocols, or conference papers. Source exclusion occurred when the topic was irrelevant (i.e., not focused on SM or SOTx populations). We excluded definitions if they were a definition that was merely a list of characteristics of SM. We also excluded extracts outlining themes or components stated generally as the background or discussion section. The secondary sources (obtained via additional bibliographic searches) did not have to refer to SOTx populations in order to be included, as we wanted to capture the original application of the cited definitions.

Screening of the definitions included in the SMART study dataset took place in Excel by two reviewers. The reviewers requested a third reviewer’s decision to gain consensus where there was disagreement about inclusion.

### 2.4. Definitions Currently in Use—Data Extraction

The following items were extracted for the analysis of definitions in this paper:

- Verbatim definitions of SM;
- Verbatim citations within definitions and definitions within the original source(s);
- Bibliographic elements were extracted from cited sources: population context and concept [40];
- Solid organ type(s);
- References to behavioural or sociological theory were recorded.

### 2.5. Bibliographic Analysis of Identified Definitions—Study Identification

Secondary cited sources were identified if they appeared within an extracted definition. Bibliographic analysis identified additional secondary source definitions composed of publications cited in the definitions we encountered initially (sources were unrestricted, e.g., books, reports, consultation documents (grey literature), and policy documents). We identified related secondary sources through several so-called generations (cited definitions).

### 2.6. Data Charting

Data charting centred on the collection and representation of data into the elements required for content analysis and conceptual analysis. Directly quoted definitions or descriptions of SM were gathered. Descriptive summaries of the application of SM approaches in interventions were also collected where there was no definition available. We did not gather whole literature review sections; slightly longer extracts were lifted when the whole article focused on definitions of SM over time.

Definitions and secondary sources were extracted into tables in Excel and Word and charted based on definition characteristics. The linkages between generations of citations were visually represented in graphic and tabulated form.

### 2.7. Data Analysis

During content analysis [37], we undertook the delineation of conceptual components. We took all verbatim definitions and created a list of individual concepts contained within them (based on the agreement between two reviewers). Next, we undertook the identification of conceptual dimensions or components as individual units of meaning; these were then condensed into cluster headings. We used the definition descriptions and textual context to understand and compare the latent meaning of codes [41], if defined in the data.

We employed analytical techniques (as a team-based participative exercise), in which we configured conceptual components of definitions into an arrangement over several iterations. Data were written onto index cards and physically arranged by the team; we also used visual representation of the arrangements of the definition components (developed in real time/ in meeting via PowerPoint). Physically representing data in this way helped us to focus on conceptual linkages we then mirrored in spatial arrangements. Therefore, we eventually turned individual components into a structured group of conceptual labels in order to describe the aspects of self-management. The structure emerged over several iterations of team-based discussion building diagrams of cluster. A consensus panel of experts was selected to assist us in refining the definition. Experts annotated an electronic version of our written draft definition. Expert roles included a professional nursing senior academic and those with experience as specialist transplant nurses (a key group in providing SM advice to patients after transplantation). Experts were existing contacts chosen selectively by the team as a preliminary step in gaining insight on the working definition.

Bibliographic analysis enabled us to understand where definitions came from and their application over time. In this way, we gathered useful information about the different SM concepts and their attributes. In addition to the data about this concept, we gathered contextual data about the application and characteristics of its application, which would also inform our critical analysis. We mapped and analysed related secondary sources through several so-called generations (cited definitions) as part of the conceptual analysis. The inclusion of a broader range of sources linked through citation tracing is a novel dimension to the aforementioned approaches. Secondary cited sources were analysed in a number of ways. First, quotations in the definitions were cross-checked with the material in the secondary sources, focusing on deviations or missing aspects. Any definitions and further cited sources were captured tracking backwards over a number of levels (or generations). Components analysed consisted of the following: theory, population, context, and publication type and study design. We assessed links between the cited sources and included definitions and patterns over time.

#### Section Critical Appraisal

There was no formal quality assessment of sources in line with the recommendations for scoping reviews [42] and other examples of definitions-focused reviews involving Concept Analysis and Critical Appraisal of the Literature [35]. The team developed a critical appraisal instrument checklist to reflect on the adequacy (appropriateness) of the definitions identified. In the assessment of the adequacy of definitions, we made assessments about definition attributes, of contexts of application, and the maturity of the concepts identified.

The first aspect involved the identification of the characteristics of key attributes, categorisation, and refinement. Judgements included elements such as the perspective of the definition, e.g., the service provider; approach to definition development; context for the definition; and applicability to the context. Whilst the second aspect considered the clarity, utility, and logic (integrity of conceptual boundaries alongside other concepts) [35].

The team incorporated these principles into our approach and developed an assessment of the adequacy of definitions, identifying what we considered central elements for a comprehensive definition. From the approach outlined by Williams et al. [35], we appraised the definition’s conceptual and theoretical background, presence of the perspective, endorsement by patient or Health Care Professional group, contexts (including temporal), and setting. We assessed the clarity and logic through criteria such as the indication of the relative importance of concepts, isolation of key concepts, relationship between concepts (process), identification of linkages to patient behaviours, and a determination about relevancy for our study definition. A criterion was used for providing an explanation of possible interventions and defining measurable outcomes. The sequential mixed method design [43] was used to inform definition attribute interpretation.

### 2.8. Ethics

No ethical approval was sought for this study as we used secondary data, and the expert feedback was considered consultation and did not require ethical approval. Contributors were anonymised.

## 3. Results

**Figure 2:**
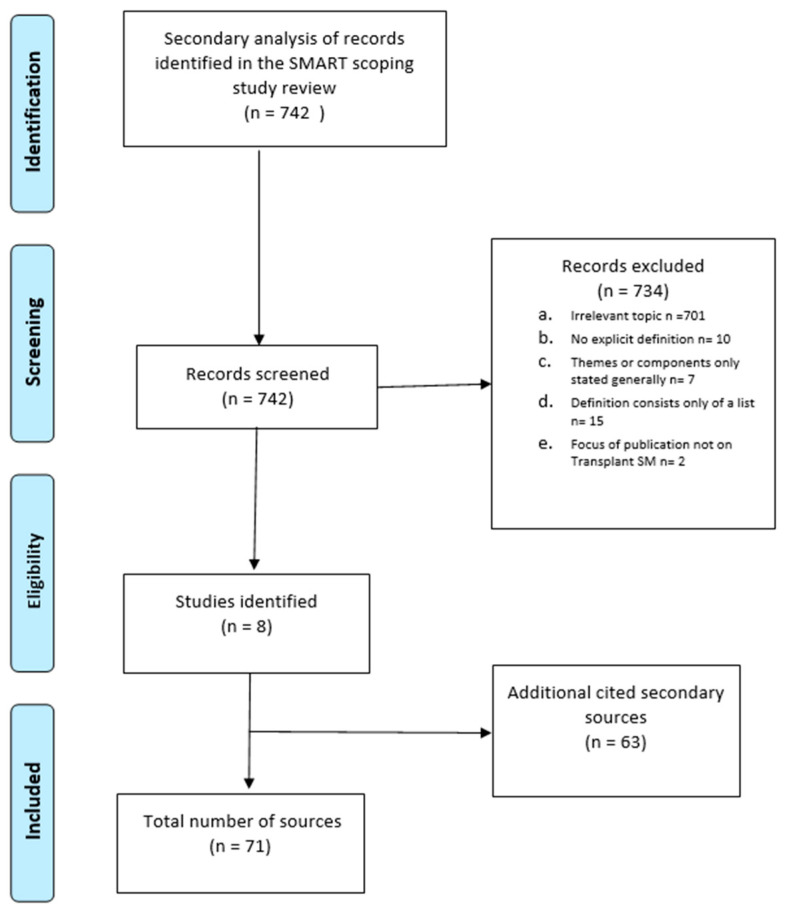
PRISMA diagram of included publications [106].

This study conducted secondary analysis of a subset of records from a SMART study scoping review (for which 34,045 records from database sources had been screened). In the context of our secondary analysis, we identified 41 records from a total pool of included 742 scoping review records contained definitions. Screening of these records resulted in eight “original” definitions [44,45,46,47,48,49,50,51]. Sixty three records were identified through secondary sources [20,21,23,25,26,28,30,31,33,34,35,52,53,54,55,56,57,58,59,60,61,62,63,64,65,66,67,68,69,70,71,72,73,74,75,76,77,78,79,80,81,82,83,84,85,86,87,88,89,90,91,92,93,94,95,96,97,98,99,100,101,102,103,104,105]. Reasons for exclusions are presented in the search results diagram below (Figure 2) based on the PRISMA statement [106].

### 3.1. Study Characteristics

Five of the definitions were published in the last three years, and the remaining were published in 2011, 2012, and 2016. A single definition focused on an aspect of SM medication adherence [44], and the remainder focused on all aspects. Definitions focused on one or multiple transplant organ types: heart [46,49,50], liver [45,48,49], kidney [44,45,49,51], pancreas [49], and lung [49] (Supplementary File S2). Amongst the definition publications, seven of the eight are quantitative studies, with one qualitative [51].

Frank-Bader et al. presented the results of an intervention on an interdisciplinary patient education programme. All the other quantitative studies were either predictive—identifying negative outcomes using patient characteristics [46,47,49] and risk factors [44,46], or described correlations between outcomes and patient symptoms/characteristics [48,49,50]. Ghadami et al.’s qualitative study explored the defining experiences of education for patients, partly, in relation to reaching a state of self-management [51]. In contrast, SM was represented as a mediating factor by Almgren et al. [50] and a correlated variable by Ko [48] (all characteristics displayed in Supplementary File S2).

### 3.2. Content Analysis of Definitions

We identified the defining attributes (conceptual components) from the eight definitions [37] (displayed in Table 1), consisting of the following: the SM process, indicators of the relative importance of aspects of SM, SM components (at various conceptual levels), examples of SM tasks or experiences, forms of support, theoretical constructs, and goals and psychological constructs or strategies. These attributes were then condensed by assigning them to conceptual attribute clusters, i.e., attributes with a similar meaning.

### 3.3. Conceptual Analysis via Bibliographic Searching

**Figure 3:**
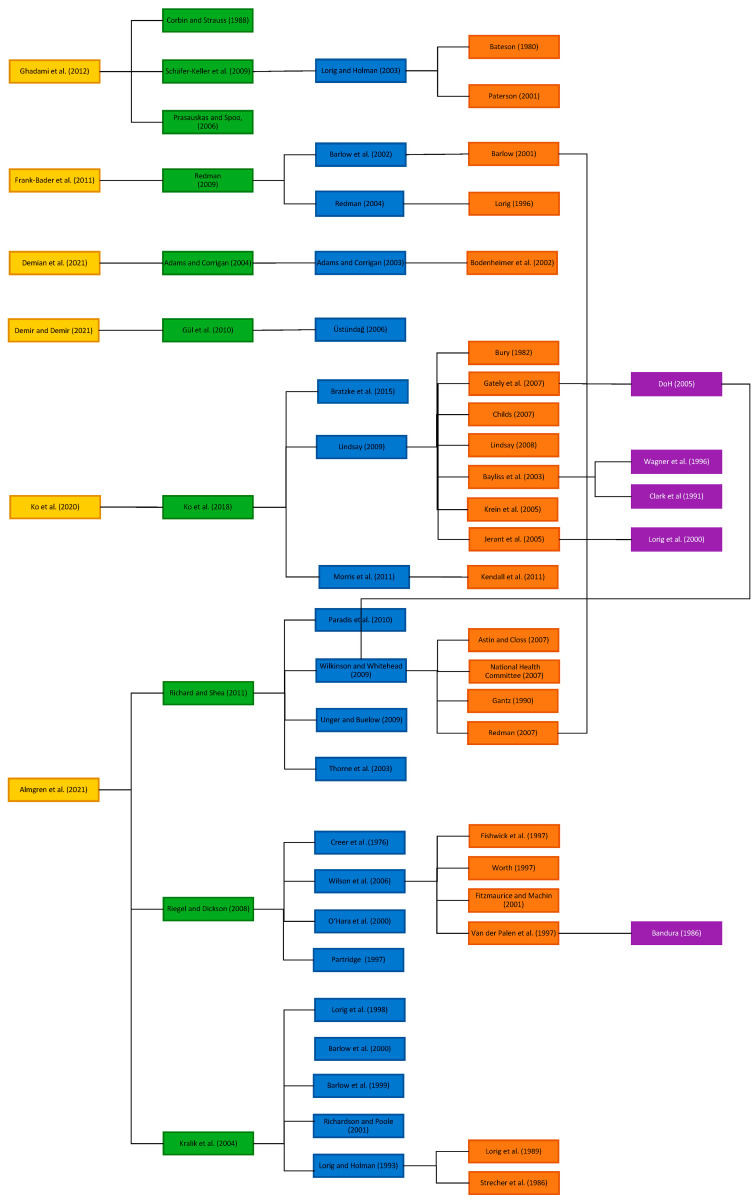
Definitions and cited secondary sources diagram [21,23,24,27,28,30,31,33,34,45,47,48,49,50,51,52,53,54,55,57,58,59,60,61,63,64,65,66,67,68,69,70,71,72,73,74,75,76,78,79,80,81,82,83,84,86,87,88,89,90,91,92,93,94,96,97,98,99,100,101,102,103,104,105].

Figure 3 represents levels of definitions and cited sources. Different colours represent the different generations of bibliographic sources. Critical analysis of secondary cited sources identified 63 unique associated publications, with 7 duplicate citations identified. Reasons for exclusions are presented in the PRISMA diagram (Figure 2). Colours correspond to the different cited secondary sources level, i.e., generations back from the original definition. In addition to the 8 definitions we identified from the scoping review, there were 20 definitions identified from secondary cited sources (although 4 are based on SOTx populations), where secondary sources were cited in more than one secondary; there are links to show this in the diagram.

Six studies cited secondary sources and two did not [44,46]. Most definitions consisted of a small number of secondary cited sources; however, 30 sources were identified from Almgren et al. [50]. Figure 3 shows the connections between definitions and Corbin and Strauss (1988) [21] theory of chronic illness management and the subsequent sources which apply this theory, such as Schäfer-Keller et al. [24,25,51].

### 3.4. SOTx SM Attributes (Cluster Headings)

We condensed the attributes into 12 cluster headings to be refined as elements of our definition in response to expert panel feedback: areas of work, moderators or facilitators, engagement in healthy behaviours, competencies, control/structure/discipline, motivation/self-efficacy, priority setting and decision making, helping/supporting/intermediates, optimising outcomes, multi-step-iterative process, external support, and cognitive/executive/memory function. These were the building blocks for our integrated definition.

### 3.5. Conceptual and Contextual Analysis of Definitions and Cited Sources

We examined the definitions in comparison to previous applications in relation to the following: deviations, omissions, and theoretical underpinning. Analysis of the eight definitions (summarised in Table 2) initially focused on the identification of missing information, which we observed in cited sources. A complete compendium of all secondary source definition extracts is provided in Supplementary File S2. Publication characteristics are tabulated in Supplementary File S3.

Amongst the included definitions, authors Patzer et al. [44] and Dalvindt et al. [46] composed their own. There were several aspects of note to emerge from the eight definitions and the first level of cited sources. Deviations in the citations of sources were identified, for example, the definition by Ghadami [51], who cite Prasauskas and Spoo [93] inaccurately. Authors opt to summarise the publication by Schäfer-Keller, referring to Corbin and Strauss framework [21] of core skills; however, they also include a phrase stating KT patients need support in the fields of knowledge, skills, and motivations, omitting dimensions such as the varying levels of importance of interaction with healthcare professionals, interactions beginning pre-transplantation. In another example, in the definition by Frank-Bader et al. [45], several aspects are absent such as inherent symptom management and physical and psychosocial consequences, as defined by Barlow [27]. The link to any outcome (or consequence) and the description of the role of self-regulation process dynamics are dropped by both Redman et al. [94] and Frank-Bader et al. [45]. A further example is the definition from Ko et al. [48] did not cite Bratzke [23] alongside Lindsay [78] and Morris et al. [86]. Demian et al. [49] refer to tasks of SM and include adherence to the medication regimen; however, the authors omit dimensions of the SM concepts present in Adams et al. [52], including self-management support and SM education.

We used a translation programme for one cited source identified in a definition ([47,70]), which contains the identical definition applied in the context of self-care power in kidney transplant patients to identify relevant text in the absence of a full text translation. However, the abstract or text for the Üstündağ [103] reference is no longer available online.

There were some minor differences in the aspects emphasised between the current and the previously cited version of definitions; for example, the first level source cited by Ko and Bratze [48] was Ko et al. [74] describing SM as iterative and ongoing, focused on prioritising care based on changing needs and conditions for chronically ill patients, whilst the definition by Ghadami et al. [51] did not emphasise self-management as a lifetime task. Another example is the definition provided by Kralik et al. [75], which differentiates between SM and coping and enablement (to minimise pain), shares in decision-making about treatment, reduces frequency of medical visits, and enjoys better quality of life (a consequence in our analysis).

Another aspect omitted from later definitions was articulated by Lorig and Holman; the authors stated “it is impossible not to manage one’s health unless one is totally ignorant of healthful behaviours”, as cited in [24] (p. 1), i.e., only the patient can be responsible for his or her day-to-day care. Another way of viewing this is the absence of specific antecedents linked to the SM concept. A final example comes from the definition by Frank-Bader et al. [45]; an examination of the second level secondary source of Barlow et al. [27] provided further clarification that “efficacious” SM is the process of monitoring of symptoms and the ability to effect psychological responses (cognitive, behavioural, and emotional).

Not only do the aspects mentioned above helped us to begin to understand the way in which defining attributes for SM changed, but the selection of certain attributes over others as definitions were adapted to SOTx populations. Demir and Demir [47] and Frank-Bader [45] referred to definitions aimed at SOTX patients, but the remaining, Almgren [50], Demian [49], and Ko and Bratzke [48], did not use SOTx SM definitions. Ghadami et al. [51] incorporated both. Omissions and deviations from the meaning and descriptions of attributes offer indications of the decision to re/shape the concept (perhaps with the exception of errors in citations identified). Citing a source which originated in a wider population base or a different context is not inherently worse than SOTx based definitions, but we must question if the basis for re-application fits the new context. The inclusion of identified theoretical backgrounds alongside cited sources sheds some light on the legitimacy of this reapplication, e.g., Demian et al. [49] omit dimensions of definitions including self-management support (which includes SM education) [52]. This source focused on the provision of educational interventions for patients by health care staff, potentially an avenue of self-management education or support which is too narrow to be lifted in entirety. Ultimately, to illuminate the question of the foundation of our included definitions, we had to look further back at the generations of cited sources.

Next, we present the analysis of all levels of cited secondary sources. There were differences in the map of the cited definitions. For example, Almgren et al. [50] contained backwards citation tracking (five levels) (see Figure 3 and Supplementary File S2). The remainder of definitions could be tracked back 2 or 3 levels (with the exception of [44,46]). There was no English translation available for sources related to Gül [69].

Thirty-four secondary sources contained a definition of self-management; of these, there were numerous publication types including a book presenting behavioural theory, a theoretical article, a paper presenting theory-generating research, a summit report of patient groups, an ethical analysis, literature reviews, conceptual overviews, empirical quantitative studies, and qualitative research articles (see Supplementary File S2). However, there were sources with alternative concepts: self-care [34,59,64,67,68,69,79,90], self-efficacy [52], coping and stress [97], and management of a chronic illness [21]. Three sources described SM as an approach to an intervention, without a definition [89,101,102]. The remainder did not include a definition.

Broader theoretical works cited included Corbin and Strauss management of chronic illness [20,21,62], Paterson’s shifting perspective model of chronic illness [92], and Bandura’s Social Cognitive Theory [53]. The description of SM by Lindsay et al. [79] cited two influential theoretical works—the sociology of health care literature by building on the concept of “biographical disruption” [58] and Corbin and Strauss “chronic illness trajectory” [21], that is, the chronic illness model comprises the management of symptoms and coping with the disease per individual on a varied trajectory according to symptoms over time. The biographical context of the patient is instrumental in understanding their experience [21]. Bury argued that the onset of chronic illness disrupted a person’s life, creating uncertainty in the domains of assumptions and behaviours; (2) the disruptions in the person’s biography and self-concept; and (3) responses to the disruption and the mobilisation of resources ([58], p. 986). In some sense, disruptions are the antecedents which are overcome by SM aspects to address the consequence of disruption and mobilisation of resources.

The patient populations in cited secondary sources used to construct the eight definitions are displayed in the Table 3. SOTx populations were present in three sources [70,94,99]. Patient groups included chronic disease populations, people with long-term conditions, and heart failure patients. However, cited secondary sources focused on a broader range of populations: people with epilepsy, chronic pain, asthma, arthritis, and people with multiple sclerosis. The sample in Wilson et al. [28] was an example of a definition combining health professionals’ (nurses, doctors, physiotherapists) perspectives with people with chronic conditions. There was an example of a non-adult sample in Creer et al. [63] and contexts less relevant to self-management in a home-based daily living context, such as primary care [30].

Findings of indicated SM definitions and sources included SOTx-specific definitions and non-chronic condition definitions at citation level 1. Beyond this, there was a mixture of general and single chronic condition-focused definitions. Definitions concentrated on general chronic disease were at level 1 and 2 as well as for arthritis (level 2 to 4) and epilepsy levels (3–4). Definitions based within studies about epilepsy patients and heart failure patients also feature in the data (levels 2–3).

### 3.6. Critique of the Adequacy of Definitions Identified

Team members applied a set of criteria developed within this project to judge the adequacy of the definitions we identified, displayed in Table 4. We developed criteria by assigning characteristics to attributes. We also added criteria we identified in our analyses phase such as patient outcomes (or in the terminology provided by Johnston et al. [1], consequences linked to the SM concept), endorsement of the definition by the patient group, and possible interventions as aspects which arose in the examination of secondary sources.

All definitions specified the population, although details were limited. There was no reference to particular patient subgroups or characteristics (age, gender, ethnicity, etc.). Almgren et al. [50] allude to social, cultural, and spiritual consequences, although this is not specifically linked to the diversity of this population and their SM experience. Three definitions contained less generalisable populations to this study definition because they focused on a single type of transplant [48,50,51], and a single study focused only on medication management [44].

The majority of definitions succeeded in specifying internal concepts, although none are defined within the SM definition. Ko et al. [48] refer to influencing factors which have a modifiable effect on SM; these can also be viewed as antecedents. The setting was not specified in any definition.

We identified references to lifespan and the length of time after transplant as evidence of a temporal aspect of definitions [44,45,47,48]. None of the definitions specified intensity of SM over time, although it may be implicit in reference to chronic illness constructions and variable symptoms [21]. Despite one mention of multimorbidity [44], none of the data mentioned the role of comorbidities. In addition, there were relatively few examples of tasks or activities or rich descriptions of concepts. Medication adherence was given as an example in three cases [44,49,51]. Demian et al. [49] alone included a patient and professional endorsed definition. Finally, a single definition mentioned family as well as HP perspectives in relation to their role as support [50], and Dalvindt constructed SM as a framework for HCP patient management; this is another way to conceptualise the so-called consequences of the SM concept [46].

### 3.7. Creation of an Integrated Comprehensive Definition

We created a comprehensive definition composed of a comprehensive set of SM attributes. One of the tools we used was a visualisation of the conceptual elements in order to arrange concepts through team discussion. During the process, we constantly referred back to the raw data and only used what was available in the identified definitions. The final definition was based on feedback from experts.

We consulted five experts from professional fields including SOTX research and practice, nursing practice and training, and health research. Issues raised by the expert group on our definition draft helped us to make some changes to the wording and enabled us to confirm gaps or weaknesses, reflecting limitations of the data. A summary of all changes is provided as Supplementary File S3. Our final definition is presented in Section 3.8. File S4 contains additional information about the expert feedback summary.

### 3.8. Final Definition for SM for SOTx Patients

Self-management (SM) for Solid Organ Transplant recipients is undertaken in order to optimise transplant outcomes and to live well. It is a multi-step and iterative process taking place over the lifetime and is therefore conceptually linked to living indefinitely with chronic illness. SM occurs in conjunction with social support systems and health professionals, who may act as external support. Practically, SM concerns different activities and tasks in three types of work (i.e., managing emotions, everyday life, and medical regimen) and requires specific competencies (knowledge, skills, and attitudes) and active engagement in healthy behaviours. Patient prioritisation of tasks and decision-making facilitated by traits of control, structure, and discipline are central characteristics, as are the moderating factors of patient motivation, self-efficacy, and cognitive function.

### 3.9. Summary of Conceptual Attributes

From the eight definitions initially identified, we assessed attributes to be both consistent with the chronic illness population experience of SM and unique to SOTx patients (summarised in Table 5).

The definition generated from this study identified a number of SOTx-specific attributes, including optimisation of transplant outcomes, active engagement in healthy behaviours, patient prioritisation of tasks and decision-making facilitated by traits, control, structure, and discipline as central characteristics, and moderating factors of patient motivation, self-efficacy, and cognitive function.

There are two attributes which could be considered applicable to all SM populations; first, that SM is a multi-step and iterative process. Secondly, SM requires specific competencies (knowledge, skills, and attitudes), assuming that specific competencies would change according to the SM group. Reference to the SOTx patient medical regimen has also been placed in this category due to the shared commonality of a medical regimen, but the difference is in terms of collaboration rather than adherence approach and the SOTx patient expertise for understanding medication and competencies is needed in relation to dosing. There are three remaining attributes which are very similar to those found in the SM list of attributes, namely, the chronic illness lens and the fact the self-management takes place over a lifetime; SM occurs in conjunction with social support systems and health professionals; and that SM concerns different activities and tasks in three types of work (i.e., managing emotions, everyday life, and medical regimen). Attributes consistent with SM more generally emphasised provision of equipment, training/rehearsal, specific clinical action plans, and regular clinical review not present in our working definition.

Conceptual attributes relevant to the SOTx population consist of antecedents (contributing factors which must be present before the occurrence of the concept) defied within the chronic illness literature base. For example, first, the patient realisation that they are living indefinitely with chronic illness as a context for SM. Secondly, SM occurs in conjunction with social support systems and health professionals. Third includes patient engagement in activities and tasks as illness work. Fourth includes patient competencies and active engagement in healthy behaviours. However, conceptualisations of attributes differ in relation to the necessity of active forms of engagement and the importance of patient traits of control and patient’s ability to create structure and discipline to facilitate prioritisation of tasks and decision-making.

Conceptualisations also included outcomes (or to use the concept analysis term, consequences [1]). These were twofold, first transplant outcomes and, more broadly, living well. It is possible that the SOTx SM field could adopt further defining terms from the general SM literature base in explaining needs or models of care despite unique requirements for optimising transplant outcomes. For instance, in a study identified within the data with elderly people with asthma, Koch et al. [107] proposed three types of self-management models; (i) the medical model—involving a passive patient, (ii) the collaborative model—whereby SM was a joint effort between them and health care professionals, and (iii) the self-agency model—where patients were experts on their own conditions through experiences. Such models help to articulate increasing levels of patient independence within a SM context and help to underline the absence of such a theory developed and tested for SOTx patients.

A relevant concept was illness work from Corbin and Strauss [21] (p. 9—Supplementary File S2). The definition belongs to the sociological theory of the illness trajectory, describing (1) course of the illness, (2) related work (and types, i.e., illness work, everyday life work, and biographical work), (3) the impact of the workers and their relationships that (4) affect the management of the course of the illness and the fate of the person who is ill ([20] pp. 225–226) requiring the combined efforts of patients, relatives, and health professionals [; developed in [21]. However, there were aspects of the framework that we did not identify in our data and represent in our definition. For example, reference to a disability framework; definitive patient strategies (opting for the phrase “patient prioritisation of tasks and decision-making”); and “handling” disability (instead conveying a wider range of emotional burden. Indeed, a revised conceptualisation of illness management describes how nurses should take a role in teaching, counselling, making arrangements, advocating, and meeting clients’ emotional needs [108] (p. 172).

Alongside the widely applied chronic illness theory, we identified other constructions of SM. For example, an SM typology for education programmes for policy-makers [73] or SM as a professional–patient joint responsibility [46], but equally, there was significant emphasis on non-adherence of the patient [44,49]. With these conceptualisations in mind, our working definition represents a patient-concordant (as opposed to compliant) perspective on SM [26].

## 4. Discussion

### 4.1. Main Findings

We identified eight definitions and 63 cited definition sources from bibliographic analysis. Bibliographic analyses of content, concepts, and contexts of application of definitions identified some limitations through deviations or omissions in meaning from earlier definitions. We also demonstrated the range of population the previous versions of definitions had been applied to, demarking the inclusion of definitions adopted from chronically ill populations generally, those from singular chronically ill populations, or even completely different populations. The theoretical works by Corbin and Strauss [21] appeared in one of our eight initial SM study definitions [51] and our secondary sources [25,30,57,78,99] and informed our proposed SM definition as an appropriate framework. Corbin advocated adjustments to the framework according to the condition or population and that nurses should create an understanding of what living with a chronic illness means for that condition in order to translate the model into practice [21] (p. 170). Therefore, we have identified some unique aspects of SM for SOTx and other aspects for future testing and consideration.

Critical appraisal of definitions indicated inadequately defined aspects such as setting, temporal dimension, concept interaction, interventions, and measurable outcomes.

Population-specific attributes included in our working definition included optimisation of transplant outcomes, active engagement in healthy behaviours, control, structure, and discipline characteristics, and moderating factors of patient motivation, self-efficacy, and cognitive function.

The review by Abtahi et al. (published after our analysis was completed) was consistent with our emphasis on management of symptoms in combination with psychosocial aspects and collaboration with others [10]. However, patient-centred care (PCC) and service delivery concepts are not represented in our data. Also, Thorne et al. argue “textbook” interventions for SM chronic disease populations were either ineffective or problematic for SOTx patients, emphasising the patient contribution to disease management decisions [101] (p. 1341).

### 4.2. Limitations

Our results are based on a comprehensive search of the recent empirical literature. Our findings are based on a systematic process designed to analyse definition content and associated concepts. The main strength of this approach is that it provides a way to configure data about definitions from a range of bibliographic secondary sources. Our results deepen the understanding about the broadening application of definitions and their limitations. The process also incorporated critical reflection to create an integrated, comprehensive definition built on the existing data.

However, this study identified limited amounts of data, so findings must be viewed with caution. The arrangement of concepts into clusters and the clusters into a definition is highly subjective. The scoping review search strategy was restricted to publications with an English or German language translation. Therefore, it is possible other definitions may exist in this field. Equally, our searches did not identify “grey” unpublished literature or books (however, our analysis of secondary sources did identify a range of contextually relevant papers, books, and reviews). We did not include patient representatives in the creation of the definition thus far.

## 5. Conclusions

The central output of this study provides a definition for SOTx patients. Findings highlight SM-facilitating factors such as patient control and the moderating factors of patient motivation, self-efficacy, and cognitive function. We looked critically at the inclusion of conceptual attributes associated with broader chronic conditions, determining that there is preferably a mix of SM-general and SOTx-specific attributes. In analysing secondary sources, this study systematically mapped and appraised definitions to understand their adoption into the SOTx literature base.

Findings informed a subsequent intervention development pilot and more broadly may contribute to future clinical practice guidance formulation for health care professionals, e.g., in decision-making support. A more comprehensive definition also provides clearer parameters for intervention development and articulates key terms and concepts to include in subsequent research and review work in this field. This review may enhance intervention design and research terminology and conceptual underpinning. This paper also represents the first step in providing a working definition that can be formally evaluated by patients and professional stakeholders to strengthen the recognition of the SM requirements of SOTx patients and to influence the provision of SM support resources.

## Figures and Tables

**Table 1:** Definitions and characteristics.

Included Publications’ Author, Year of Publication, Title	Methodology	Summary of Study Methods and Results	Definition Extract within Included Publication	Defining Attributes (Conceptual Component Codes) from 8 Definitions
Almgren et al., 2021 [50] Self-efficacy, recovery and psychological wellbeing one to five years after heart transplantation: a Swedish cross-sectional study	Cross sectional Obs (Quant)	Methods: cross sectional study with 79 HTx; instrument: German version of the self-efficacy for managing chronic disease 6-item scale (SES6G). Results: level of self-efficacy was high, fully/partly recovered HTX; overall good wellbeing in population. Discussion: self-efficacy is about balancing expectations; self-efficacy is a mediator for self-management	The success of transplantation partly rests on the self-management ability of the heart transplant recipient (HTR), in conjunction with family and transplant professionals to manage symptoms, treatments, lifestyle changes and psychosocial, cultural and spiritual consequences. After HTx self-management is mainly constituted by the ability and process that the HTR uses in conscious attempts to gain control of his or her everyday life with a new heart rather than being controlled by it [33].” “Self-management focuses on the activities people carry out in order to create structure, discipline and control in their lives [50].” (p. 35)	In conjunction with family and transplant professionals > To manage symptoms, treatments, lifestyle changes, and psychosocial, cultural and spiritual consequences > Is mainly constituted by the ability and process > To gain control of his or her everyday > Focuses on the activities to create structure, discipline, and control in their lives
Demian et al., 2021 [49] Negative affect and self-agency’s association with immunosuppressant adherence in organ transplant	Systematic review—meta-analysis	Methods: meta-analysis. Results: 50 studies included, increased NA is associated with worse adherence, high self-agency associated with good adherence. Discussion: different adherence measurement methods applied in the studies; cultural effect on association	“Living with a transplant requires a high degree of self-management, defined as ‘the tasks [one] must undertake to live well with one or more chronic conditions’ (Adams et al., 2004, p. 57) [70] and includes adherence to the medication regimen” (p. 90)	The tasks [one] must undertake > To live well > Includes adherence to the medication regimen
Demir and Demir, 2021 [47] Effects of illness perception on self-care agency and hopelessness levels in liver transplant patients: a descriptive cross-sectional study	Descriptive /exploratory/ obs(quant)	Methods: descriptive cross-sectional method, “Patient Identification Form (PIF)”, the “Brief Illness Perception Questionnaire (B-IPQ)”, the “Self-Care Agency Scale (SCAS)”, and the “Beck Hopelessness Scale (BHS). Results: 120 Ltx correlation between BHS and B-IPQ, mean hopelessness scale scores. Discussion: high negative illness perception, mean sores of self-efficacy, no correlation between self-efficacy and illness perception→but LTx feeling stronger; participation in care	“After the transplant, it is necessary to increase the self-care ability of the patient to take an active role in protecting, improving, and raising their own health, to continue their daily life activities, and to ensure transition to normal life as soon as possible (Gül et al., 2010)” (p. 474)	Increase the self-care ability > To take an active role in protecting, improving, and raising their own health > Continue their daily life activities > Ensure transition to normal life as soon as possible
Dalvindt et al., 2020 [46] Symptom occurrence and distress after heart transplantation: a nationwide cross-sectional cohort study	Descriptive/exploratory/ observational (quant)	Methods: wellbeing instruments→Psychological General Well-Being (PGWB), Organ Transplant Symptom and Well-being Instrument (OTSWI). Results: 79 HTx; most common symptoms: trembling hands, decreased libido; sociodemographic factors, more symptoms when not working; with poor psychological wellbeing, living alone, depended on follow up. Discussion: fatigue is strongest predictor	“Self-management has been adopted by transplant professionals as a framework for efficient support to transplant recipients in managing their chronic condition, namely the transplantation” (p. 2)	A framework for efficient support > Managing their chronic condition
Ko and Bratzke, 2020 [48] Cognitive function in liver transplant recipients who survived more than 6 months	Secondary data analysis (quant)	Methods: secondary data analysis with Monreals Cognitive Assessment, and Health Education Impact Questionnaire (heiQ), and the Basel Assessment of Adherence with Immunosuppressive Medication Scale (BAASIS). Results: 107 Ltx; More than half of the recipients had global cognitive impairment. Age was associated with significant differences in global cognitive function. Discussion: SM and cognitive function are somehow related	“Liver transplant recipients ability to self-manage, which for this study is defined as ‘an iterative process of priority setting and decision making for the practical management of an illness’ is likely influenced by cognitive function, especially memory and executive function [74]” (p. 1)	An iterative process > Priority setting > Decision-making > Practical management of an illness > Cognitive function, especially memory and executive function
Patzer et al., 2016 [44] Medication understanding, non-adherence, and clinical outcomes among adult kidney transplant recipients	Descriptive/exploratory/ observational (quant)	Methods: in-person interviews about medication knowledge, regimen use, medication adherence. Results: 99KTx high percentage (35%) of non-adherence to immunosuppressive medication. Discussion: higher number of medications, lower health literacy level, longer time after Tx leads to medication non-adherence	“Medication self-management for transplant recipients is a multistep process by which organ transplant recipients take their medication. The patient must first fill the prescription, and then, the patient should be able to correctly name, identify, and understand the medication. The third step is organization of multiple medications into the appropriate dosing frequency. Next, actually taking the medication at the correct dosage is essential. For those who are on complex or multiple medications, monitoring medication changes is essential. Finally, patients must sustain medication behaviors indefinitely to achieve medication self-management” (p. 1295)	Multi-step process > Monitoring medication changes is essential
Ghadami et al., 2012 [51] Patients’ experiences from their received education about the process of kidney transplant: a qualitative study	Qualitative	Methods: qualitative study with content analysis approach with 18 participants. Results: need for educational experiences at the beginning and end of transplantation; personal struggle to increase awareness to reach self-management and transplanted kidney preservation. Discussion: demand for efficient education to achieve the level of decision-making and problem-solving; demand for encouragement	“Renal transplant recipient self-management can be divided into the same components as used for other chronic illness populations: (1) management of the medical regimen, (2) management of the emotions and (3) management of the new life roles [20]. Since KT patients need support in fields of knowledge, skills and motivations, [99] they should acquire awareness, skills and attitudes as well as adequate resources to attain healthy behaviours in order to feel responsible” (p. 158)	Can be divided into the same components as used for other chronic illness populations > Management of the medical regimen > management of emotions > Management of the new life roles > Need support in fields of knowledge, skills, and motivations > Acquire awareness, skills, and attitudes > To attain healthy behaviours in order to feel responsible
Frank-Bader et al., 2011 [45] Improving transplant discharge education using a structured teaching approach	Best/clinical practice article	Methods: development of standardised teaching process to Ktx/LTx, strategies to encourage patient and families. Results: patient and nurses’ satisfaction with teaching process. Discussion: structured learning process helped to minimise the amount of information at one time	“Redman (2009) [94] has posited that self-management is also essential for transplant patients because, although transplantation itself is an acute intervention, living with the transplant is a chronic condition. Therefore, patients must have the self-efficacy and knowledge and skills to manage their own care over a lifetime” (p. 332)	>Essential > Acute intervention > Chronic condition > Must have self-efficacy > Must have knowledge < Must have skills > Over lifetime

**Table 2:** Analysis of definitions and cited secondary sources (comparison of initial set of 8 definitions’ content with cited sources).

Reference and Year of Publication	SOTx Population Focus in Cited Sources?	Missing Aspects not Present in Our Identified Definitions Found in Level 1 Secondary Sources	Deviations in Summary	Theoretical Basis Identified in 8 Definitions
Almgren (2021) [50]	No	1 Richard and Shea 2011 [33] 2 Kralik 2004 [75] Richard and Shea 2011 [33], Wilkinson and Whitehead (2009) [34]—second generation cited source, who also included community in the collaborative aspect of SM	Richard and Shea 2011 [33] is citing another (original) source: Thorne 2003 [101]	None
Demian (2021) [49]	No	Demian et al. 2021 [49] omit dimensions of definitions from [69,74] Adams 2004 [70] reference—including self-management support (which includes SM education)	None identified	(Adams et al., 2003) [71] Self-management support: the systematic provision of education and supportive interventions by health care staff to increase patients’ skills and confidence in managing their health problems, including regular assessment of progress and problems, goal setting, and problem-solving support
Demir and Demir (2021) [47]	Yes	1 No full translation available for Gül 2010 [69] Üstündağ (2006) [103] (a second generation cited source—no full text available)	None identified	Self-care ([69] renal transplantation discharge education), concepts: the patient to protect and improve their own health, to take an active role in upgrading, daily life self-care, to maintain their activities, to increase the ability to live a normal life as soon as possible
Dalvindt et al. [46]	N/a	Authors composed their own definition	Not applicable	None
Ko and Bratzke 2020 [48]	No	Ko 2018 [74] Source cited in Ko et al. 2018 is Bratzke et al. 2015 [23] with Lindsay 2009; Morris et al. 2011 [78,86]	None identified	Self-management—iterative process, ongoing process, prioritising care based on changing needs and conditions
Patzer (2016) [44]	N/a	Authors composed their own definition	Not applicable	None
Ghadami (2012) [51]	1 No 2 Yes 3 No	1 Corbin JM, Strauss 1988 [21] 2 Schäfer-Keller et al. 2009 [99] Concept missing from summary: “It is impossible not to manage one’s health. The only question is how one manages.” Self-management is a lifetime task. From Lorig and Holman (2003) p. 1 [24] Schaffer-Keller (2009) summarise important aspects of the cited model (from Corbin and Strauss 1988): (i) kidney transplant recipient self-management includes managing a medical regimen, emotions, and (new) life roles; (ii) SOTx may affect the patient’s family and/or community and should significantly influence interaction with healthcare professionals; (iii) this may begin pre-transplantation; (iv) specific aspects assuming varying levels of importance at each stage; (v) core skills [10,13] deemed reasonable for kidney recipients to have or acquire p. 111 3 Prasauskas and Spoo 2006 [93] Home health care management practice and delivery of information to improve patient outcomes	3 Prasauskas and Spoo, 2006 [93] cited, however, this does not accurately summarise elements in the publication: use a teach-back technique for addressing home care for the elderly if managing their own care. The paper emphasises responsibility of a clinician and delivery of information. -This paper is about health literacy, not self-management -Likely the associated source is Schäfer-Keller 2009 [99], who talk about control	Corbin JM, Strauss 1988 [21], patient education
Frank-Bader (2011) [45]	Yes	Redman 2009 [96] Inherent symptom management, physical and psychosocial consequences (defined by Barlow (2002) [27] within citation) are absent	Barlow (2002) [26], definition within Redman (2009) [94] not cited	None

**Table 3:** Populations for cited secondary sources.

Population		SOTX		C/I	Gen.					C/I	Single					Non-C/I	
	SOTx	SOTx—kidney	1	2	3	4	5	6	7	8	9	10	11	12	13	14	15
Source level 1	●	●●	●													●	●
Source level 2			●●●●●	●●●					●●●●●				●●	●●●	●		
Source level 3			●●●●●●●●●●	●	●	●	●●	●●	●●	●			●	●		●	
Source level 4			●●					●●	●●		●	●					

● Represents a secondary cited source publication; SOTX, C/I (chronic illness). Gen. C/I (chronic illness general population). single non-C/I (single chronic illness groups). Key to columns representing population groups in definitions: 1. chronic disease; 2. multimorbidity; 3. people with long-term conditions; 4. health professionals who care for patients with LT conditions; 5. diabetes and comorbidities; 6. asthma; 7. arthritis; 8. people with multiple sclerosis; 9. COPD; 10. oral anticoagulation; 11. epilepsy patients; 12. heart failure patients; 13. chronic pain; 14. all nursing populations; 15. adult low health literacy.

**Table 4:** Mapping reported key features of a definition.

Definition Component	[50]	[46]	[49]	[47]	[51]	[45]	[48]	[44]
Defines population	●	●	●	●	●	●	●	●
Defines conceptual/theoretical approach	●	●	○	○	●	●	○	○
Defines settings of SM	○	○	○	○	○	○	○	○
Isolates key concepts	●	○	●	●	●	●	●	●
Defines key concepts	●	○	○		●	○	●	●
Indicates relative importance of concepts	○	○	○	●	○	●	○	●
Explains relationship between concepts (process)	●	○	○	○	○	○	●	●
Linkages to patient behaviours	●	○	○	●	●	●	●	●
Contextualised temporally	○	○	○	●	○	●	●	●
Defines various relevant persons/perspectives	●	○	○	○	○	○	○	●
Inclusion of HCP perspective	●	●	○	○	○	○	○	○
Provides an explanation of possible interventions	○	○	○	○	○	●	○	○
Defines measurable outcomes	●	○	○	●	●	○	○	○
Inclusion of or reference to defining elements endorsed by patient or HCP group	○	○	●	○	○	○	○	○
Relevancy for our study to create a definition for the entire SOTX population for all SM tasks	○ Heart transplant recipient	●	●	●	○ Renal transplant recipient	●	○ Liver transplant recipients (from first 6 months)	○ (Medication only)

Definition component met ●. Definition component not met ○.

**Table 5:** Classification of SOTx working definition features.

Attribute in Our Working Definition of SM for SOTx	Type of Population Attribute	Associated Attribute within SM General Population [2]
To optimise transplant outcomes	SOTx population only	-
Active engagement in healthy behaviours	SOTx population only	-
Patient prioritisation of tasks and decision-making facilitated by traits	SOTx population only	-
Control, structure, and discipline are central characteristics	SOTx population only	-
Moderating factors of patient motivation, self-efficacy, and cognitive function	SOTx population only	-
Medical regimen	SOTx population only	-
It is a multi-step and iterative process	Attributable to SOTx and possibly SM general population	
Requires specific competencies (knowledge, skills, and attitudes)	Attributable to SOTx and possibly SM general population	Information about condition and/or its management Training/rehearsal for psychological strategies,
Taking place over the lifetime and is therefore conceptually linked to living indefinitely with chronic illness	Attributable to general SM population	
SM occurs in conjunction with social support systems and health professionals	Attributable to general SM population	Social support and lifestyle advice and support Provision of/agreement on specific clinical action plans, regular clinical review
SM concerns different activities and tasks in three types of work (i.e., managing emotions, everyday life, and medical regimen)	Attributable to general SM population	Training/rehearsal for practical self-management activities Monitoring of condition with feedback, practical support with adherence—medication or behavioural Provision of equipment, training/rehearsal for everyday activities

## Data Availability

The datasets used and/or analysed during the current study are available from the corresponding author on reasonable request.

## Supplementary Materials

The following supporting information can be downloaded at: https://www.mdpi.com/article/10.3390/nursrep14020073/s1, File S1: RAMESES report checklist, File S2: Additional information about cited secondary sources’ definition extracts; File S3: Secondary source characteristics, and S4: Additional information about expert feedback summary.

## Public Involvement Statement

No public involvement in any aspect of this research.

## Guidelines and Standards Statement

This manuscript was drafted using the RAMESES guidelines [39] for reporting meta-narrative review research.
