# Supplementary material for: Defining Self-Management for Solid Organ Transplantation Recipients: A Mixed Method Study
Source: Nurs Rep. 2024 Apr 17;14(2):961–87. doi: 10.3390/nursrep14020073 (PMC11036239; doi:10.3390/nursrep14020073)
Supplement: Supplementary file 1 [file nursrep-14-00073-s001.zip › S1.pdf]

Supplementary File S1 (S1): RAMESES -Realist And MEta-narrative Evidence Syntheses: Evolving Standard (2013) Reporting checklist for narrative reviews [39]

Section of paper	RAMESES item	Comment	Inclusion of item
Title	1 In the title, identify the document as a meta-narrative review or synthesis	We have labelled the methodological approach of the review as a mixed method<br>- A mixed method review of definition content, concepts and context	N/A
Abstract	2 While acknowledging publication requirements and house style, abstracts should ideally contain brief details of: the study's background, review question, or objectives; search strategy; methods of selection, appraisal, analysis, and synthesis of sources; main results; and implications for practice		Y
Introduction- Rationale for review	3 Explain why the review is needed and what it is likely to contribute to existing understanding of the topic area		Y
Objectives and focus of review	4 State the objective(s) of the review and/or the review question(s). Define and provide a rationale for the focus of the review.		Y
Method-<br>Changes in the review process	5 Any changes made to the review process that was initially planned should be briefly described and justified.	Development of critical appraisal of definitions (criteria) and integrated definition emerged iteratively throughout the analysis process<br>No other changes – stated in manuscript	Y
Rationale for using meta-narrative review	6 Explain why meta-narrative review was considered the most appropriate method to use.	The methodological approaches we chose combined analysis of content of definitions and synthesis of concepts.	N/A
Evidence of adherence to guiding principles of meta-narrative review	7 Where appropriate show how each of the six guiding principles (pragmatism, pluralism, historicity, contestation, reflexivity, and peer review) have been followed	Meta narrative principle were not explicitly stated in analysis or discussion sections as we employed principles of content analysis, conceptual analysis and critical appraisal criteria. In addition, we collected publication characteristics to understand contribution of findings.<br><br>However, in the discussion we explore:<br>-highlight where narratives from sources are in agreement.<br>- influential theory (through a historical lens)<br>- definitions are mapped over time,	N/A

		-we provide areas of contestation in aspects of definitions according to features such as population,<br>-we critically reflect on secondary sources, we analyse study characteristics and if theory was empirically-driven,<br>-collated publication types and characteristics such as conceptual focus to determine relevancy<br>-presented narratives of peer reviewed overviews of the literature in reference to wider literature in the discussion section.	
Scoping the literature	8 Describe and justify the initial process of exploratory scoping of literature.	Reference to scoping review protocol from broader study and description of identification of definitions.	Y
Searching processes	9 While considering specific requirements of the journal or other publication outlet, state and provide a rationale for how the iterative searching was done. Provide details on all the sources accessed for information in the review. Where searching in electronic databases has taken place, the details should include (for example) name of database, search terms, dates of coverage and date last searched. If individuals familiar with the relevant literature and/or topic area were contacted, indicate how they were identified and selected	Secondary sources identification described. Database searches described.	Y
Selection and appraisal of documents	10 Explain how judgements were made about including and excluding documents	Inclusion criteria for definitions study provided. Inclusion criteria for broader review cited. Document types also provided.	Y
Data extraction	11 Describe and explain which data or information were extracted from the included documents and justify this selection	Definitions data extraction described	Y
Analysis and synthesis processes	12 Describe the analysis and synthesis processes in detail. This section should include information on the constructs analysed and describe the analytic process.	Analytical process described for creating integrated definition and adequacy of definition criteria	Y
Results<br>Document flow diagram	13 Provide details on the number of documents assessed for eligibility and included in the review with reasons for exclusion at each stage as well as an indication of their source of origin(e.g. from searching databases and reference lists). You	Search PRISMA flow diagram included	Y

	may consider using the example templates (which are likely to need modification to suit the data) that are provided		
Document characteristics	14 Provide information on the characteristics of the documents included in the review.	Provided in tables and supplementary files	Y
Main findings	15 Present the key findings with a specific focus on theory building and testing	Integrated definitions and critical analysis of secondary sources provided with specific explanation of theory	Y
Discussion Summary of findings	16 Summarize the main findings, taking into account the review's objective(s), research question(s), focus, and intended audience(s).	Main findings summarised	Y
Strengths, limitations, and future research directions	17 Discuss both the strengths of the review and its limitations. These should include(but need not be restricted to) (a) consideration of all the steps in the review process and (b) comment on the overall strength of evidence supporting the explanatory insights which emerged. The limitations identified may point to areas where further work is needed.	Section within paper on strengths and limitations of review	Y
Comparison with existing literature	18 Where applicable, compare and contrast the review's findings with the existing literature (e.g. example, other reviews) on the same topic	Discussion of findings in relation to wider literature and secondary sources which provided historical/conceptual overview	Y
Conclusion and recommendations	19 List the main implications of the findings and place these in the context of other relevant literature. If appropriate, offer recommendations for policy and practice.	Implications for policy and practice stated- in particular, further development of definition	Y
Funding	20 Provide details of funding source (if any) for the review, the role played by the funder (if any) and any conflicts of interests of the reviewers.		Y
