# Supplementary material for: Defining Self-Management for Solid Organ Transplantation Recipients: A Mixed Method Study
Source: Nurs Rep. 2024 Apr 17;14(2):961–87. doi: 10.3390/nursrep14020073 (PMC11036239; doi:10.3390/nursrep14020073)
Supplement: Supplementary file 1 [file nursrep-14-00073-s001.zip › S2.pdf]

Supplementary File S2 (S2): Cited secondary sources definition extracts characteristics

SM definition/ Descriptive summary of application of SM intervention / other concept/ none	Source and definition extract
**Definition of self-care**	Gül (2010) [69]<br>"*After the transplant, the patient to protect and improve their own health and to take an active role in upgrading, daily life self-care in order to maintain their activities to increase the ability to live a normal life as soon as possible transition should be ensured in a timely manner (5).*" (translated in Deepl) p.8<br><br>Üstündağ H. (2006) [103]– Translation not available
**Definition**	Ko (2018) [74]<br>We have chosen to define self-management as an iterative process of priority setting and decision making for the practical management of an illness, using the definition of Bratzke et al. (2015) [23].<br>… The authors of this review and multiple qualitative studies have defined self-management in the context of multiple chronic conditions as an ongoing process of prioritizing care based on changing needs and conditions Bratzke et al., 2015 [23]; Lindsay, 2009 [79]; Morris et al., 2011 [86]<br><br>Bratzke et al. (2015) [23]<br>Lindsay, S. (2009) [79]<br>Morris et al. (2011) [86]
**Definition**	Bratzke et al. (2015) [23]<br><br>*While some definitions of self-management include emotional coping activities (Kralik et al., 2004), for the purpose of this review, self-management is defined as behaviors and activities an individual employs for the practical management of an illness, such as taking medications, and managing physical or functional effects of the illness* p.745
**Definition**	Lindsay (2009) [79]<br><br>Abstract<br>This study adds to the sociology of health care literature by building on Bury's (1982) concept of "biographical disruption" and Corbin and Strauss "chronic illness trajectory" and examining the extent to which they apply to patients managing multiple chronic conditions.<br><br><u>The Self-management of Chronic Disease</u>

	Self-care is a major component of chronic disease management because the majority of illness management takes place outside of formal care (Gately et al. 2007). Having multiple chronic illnesses can influence a patient's ability to self-manage their health (Childs 2007; Lindsay 2008). Although little is known about the process of self-managing multiple chronic illnesses, several studies have examined the barriers that patients experience in doing so. For example, barriers often include interaction effects of conditions and medications (Bayliss et al. 2003), difficulty following recommended exercise and dietary plans (Krein et al. 2005), depression, fatigue, poor communication with physicians, lack of social support, pain and physical symptoms, financial problems, lack of awareness, and transportation problems (Jerant et al. 2005). The burden for self-management is heavy for people with multiple chronic conditions.p984<br><br>Bury (1982) [58]<br><br>Gately et al. (2007) [68]<br><br>Childs (2007) [60]<br><br>Lindsay (2008) [78]<br><br>Bayliss et al. (2003) [59]<br><br>Krein et al. (2005) [76]<br><br>Jerant et al. (2005)<br><br>Corbin and Strauss (1991) [62].
**Definition of management of chronic illness:**	Corbin and Strauss (1991) [62].<br><br>*The management of chronic illness is not just a matter of controlling symptoms, or living with a disability or adapting to the psychological or adapting to the changes that long-term incurable illness bring to the lives of the afflicted individuals and their families. All of these and more. P. 155*<br>*The Trajectory framework is a conceptual model based on the idea that chronic conditions have a course that changes and varies over time.*
**None**	Bury (1982) [58]
**Self-management support definition**<br><br>**SM and self-care education with a self-care definition**	Gately et al. (2007) [68]<br><br>*Self-care has been defined as: 'the care taken by individuals towards their own health and wellbeing: it comprises the actions they take to lead a healthy lifestyle; to meet their social, emotional and psychological needs; to care for their long-term condition; and to prevent further illness or accidents' (Department of Health, 2005b).* p.934-5… Department of Health. (2005b). Self-care—A real choice: Self-care support—A practical option. London: Crown Copyright<br>(Policies for managing long-term conditions increasingly envisage their care being based around three tiers: case management for patients with multiple, complex conditions; disease management for patients at some risk, through guideline based programmes in primary care

(Department of Health, 2005a; Wagner et al., 2001); and self-care support for low risk patients (70–80% of those with long-term conditions)).

One of the aims of top-down policy programmes of self-management support has been to contribute to more effective chronic disease management. Self-management education has also been identified as having a key role to play in utilisation reduction and containing health care spending (Department of Health, UK, 1999, 2005b).
