# Supplementary material for: Defining Self-Management for Solid Organ Transplantation Recipients: A Mixed Method Study
Source: Nurs Rep. 2024 Apr 17;14(2):961–87. doi: 10.3390/nursrep14020073 (PMC11036239; doi:10.3390/nursrep14020073)
Supplement: Supplementary file 1 [file nursrep-14-00073-s001.zip › S3.pdf]

Supplementary File S3 (S3): Secondary source characteristics

Focus of study/publication	SM definition/ Descriptive summary of application of SM intervention / other concept/ none	Source	Population	Context/setting of study or publication	Concepts examined	Publication: type	Study design & methods, sample
The aim of the study was to evaluate the self-care agency and determine the affecting factors in renal transplant patients.	Definition of self-care	[69] Translation not available<br>[103] FT not available	Kidney SOTx	[69] Turkish<br><br>Patients who had renal transplant in a university hospital.	[69] self-care agency (component of Orem's Self Care Deficit Nursing Theory)	Journal article, (quantitative)	Descriptive cross-sectional study (N50)
Unknown	Unknown	[103]–FT not available	Unknown	Unknown	Unknown	Unknown	Unknown
To examine how self-management has been operationalized in the presence of multiple chronic conditions	Definition	[74] Citing [23,79,86]	Adults with at least two or more chronic illnesses (including mental illnesses)	(Narrative review)<br><br>Countries NR	SM	Journal article,	Narrative review

				Assessment of service delivery and telehealth interventions			
To synthesize current research findings related to self-management, in order to better understand the processes of priority setting and decision-making among adults with multi-morbidity	Definition	[23]	Adults with multi-morbidity	A narrative review Studies from Europe, 8 from USA<br><br>Patient experience studies in primary care or home/community settings	SM	Journal article	Narrative review
To develop a better understanding of how people with multiple chronic illnesses prioritize their various conditions<br>The overriding aim of this nine-month trial was to test whether<br>facilitated access to an Internet health portal could improve the capacity<br>of men and women with heart disease to manage their own heart conditions.	Definition	[79] Citing [58–60,62,68,72,76,78]	Patients with heart disease	home/community settings	Study examines the concepts of “chronic illness trajectory” and “biographical disruption” to examine how patients self-manage	Journal article	qualitative mixed methods- Questionnaires, focus groups, and Internet forums (N53)
To explain the model of chronic illness management based upon the trajectory framework and implications for nursing	Definition of management of chronic illness:	[62]	Patients with chronic conditions	(Non-empirical) Theory intended for nursing practice	Theoretical concerns of the sociology of work are addressed as	Journal article	theoretical paper

(non-empirical)					well as the substantive issues of managing chronic illness. Model of chronic illness management based upon the trajectory framework. Including trajectory management, biography, everyday life activities		
To explore a limited range of theoretical and empirical issues *thrown up* by the study of chronic illness	None	[58]	Rheumatoid arthritis patients	England<br><br>Outpatient clinic	Chronic illness as a disruptive event	Journal article	qualitative study and theoretical paper (N30)
To illuminate the nature and the processes of utilisation which accompany self-management skills-training by exploring the way in which patients related to services prior and subsequent to participation in a self-care intervention.	Self-management support definition<br><br>SM and self-care education with a self-care definition	[68] Citing [64]	Patients recruited across a range of chronic conditions	England<br><br>Qualitative study of health service use in context of RCT-intervention was EPP is a group-based intervention delivered in community. The programme is based on self-efficacy theory (Bandura, 1977)-	self-management education- including biographical and social context	Journal article	longitudinal qualitative study (N23)

				aims to improve self-efficacy			
Self-care support	Self care support definition	[64]	People with long-term conditions	England, UK<br><br>NHS services and at home	Self-care	Policy document	N/A
To provide an overview of care and conceptual models for patients with diabetes and comorbidities	None	[60]	Patients with diabetes and co morbidities	Non empirical	Complications, Self care	Journal article	literature and theory review
To explore the barriers experienced in self-managing it. Questionnaires, focus groups, and Internet forums were analysed to explore Obstacles in self-managing CHD.	None	[78]	Elderly heart disease patients from a deprived, inner city area	Manchester, UK<br><br>sample derived from larger study primary care database<br><br>Community / at home setting	Inequalities in managing chronic illness	Journal article	qualitative study, Questionnaires ,30 focus groups, discussion forums, and Internet log data (N108)
To identify perceived barriers to self-care among patients with comorbid chronic diseases.	Definition	[59] Citing [61,104]	Patients with Comorbid Chronic Diseases	Colorado, USA<br><br>(Urban) primary care practice recruitment for perspectives about at home care	Self-care	Journal article	qualitative study, semi-structured interviews (N16)
Reviewed literature for changes to traditional care systems that would improve care and to improve and refine the Milbank article model-	None	[104]	Patients with chronic illness	Authors asked 75 experts in chronic illness management to review the revised CCM model.	Milbank Chronic Care Model	Journal article	literature review

Review of SM	Definition	[61]	Older adults with chronic disease and in general	At home SM from studies reporting on a range of SM tasks e.g. smoking cessation, adapting to work, using medications	SM,<br>At home management and psychosocial coping, self regulation and control,<br>Bandura Social Cognitive Theory (1986) theory<br>Self care	Journal article	overview of theory and research
To examine<br>whether and how chronic pain affects diabetes self-management.	None	[76]	Patients with diabetes and chronic pain	USA<br><br>Patients receiving care through the Department of Veterans Affairs (VA). Focus at home self-care	SM<br>Self-care	Journal article	Quantitative cross sectional study (N993)
Authors conducted 10 focus groups to inform development of Homing in on Health (HioH), a home delivery variant of the Chronic Disease Self-Management<br>Program (CDSMP)	Definition	[72]<br>Citing<br>[80]	Chronic disease<br>(Diabetes 37 (69)<br>Arthritis 31 (57)<br>Depression 24 (44)<br>CHF 21 (39)<br>Asthma 13 (24)<br>Other)	USA<br><br>Development of an intervention (community)	active self-management	Journal article,	Qualitative study to inform an intervention, focus groups (N54)
Self-management for chronic illnesses- support guidance for SM skills for patients	Definition	[80]	Chronic conditions (Heart Disease, Arthritis, Stroke, Diabetes, Asthma,	Non-empirical<br>USA<br><br>Patient guidance	SM skills	Book,	non-empirical

			Bronchitis, Emphysema and Others)				
To examine what influences self-management priorities for individuals with multiple long-term conditions and how this changes over time.	Definition	[86] Citing [73]	Participants with more than one chronic condition: irritable bowel syndrome; chronic obstructive pulmonary disease and diabetes.	North west, UK<br><br>SM at home	SM<br><br>dynamic prioritization of conditions	Journal article,	qualitative study, semi-structured interviews (N21)
Authors used a corpus of data collected over the past 5 years to explore the assumptions that underpin common usage of the term self-management among policy-makers, professionals and consumers.	Definition	[73]	None specified-theory-based paper	Australian policy and international research	SM - Self-managing self-management	Journal article	overview of literature (including definitions)
To identify strategies that can be used by communities to help patients manage their condition(s) while leading active and productive lives	Definition	[70] [71]	Patients with chronic conditions	Non-empirical<br><br>Summit in 2004 of the IOM; Institute of Medicine, USA- Chronic illness communities contributed	SM	Summit report	discursive summary of strategies
To establish priority areas for policy and research	Definition	[71]	Community sample - Asthma and heart failure patients	Non empirical Report was compilation of secondary evidence	SMS	Report-	summary of Committee on Identifying Priority Areas for Quality Improvement

							(reference to secondary evidence)
To present innovations in SM education in primary care	Definition	[30] Citing [109]	Patients with chronic conditions	Literature review<br><br>Studies contained populations from studies and RCTs on asthma, diabetes, arthritis, chronic conditions generally<br><br>USA; Primary care	self-management; collaborative Care, self-efficacy in SM; patient physician partnership	Journal article	evidence summary (RCT focus
To understand the way in which people who lived with chronic illness constructed the notion of self-management.<br>Population: Patients with Arthritis	Definition	[75] Citing [55,56,82,84,97]	Patients with a chronic illness	Australia, home setting	SM and evaluation of SM Programmes	Journal article	qualitative research, autobiographical study N9
To examine the 12-year history of the Arthritis Self-Management<br>Studies.	Definition	[82] Citing [83,100]	Patients with arthritis and pain	Review of secondary evidence<br>And intervention results<br><br>USA<br><br>Patients report on at home pain	SM<br>Self-efficacy<br>Patient education<br>Control<br>Cost effectiveness	journal article	quantitative evaluation of an intervention, self-administered questionnaires, 3 randomised studies of different associations)<br>N707 N107 N 224

To develop a self-efficacy scale for people with arthritis	None	[83]	People with arthritis	USA<br><br>Measures assessed within a self management course	Self-efficacy<br>SM	journal article,	quantitative evaluation of a measure, N54
To facilitate a clearer understanding of both the concept and its relevance for health education research and practice	None	[100]	Patients requiring behavioural change intervention in cigarette smoking, weight control, contraception, alcohol abuse and exercise.	Secondary evidence<br><br>Behavioural lifestyle interventions	Self-efficacy<br>Bandura's Health education<br>Social Learning Theory	Journal article	literature review of empirical studies (including survey, intervention and experimental design)
Determine strengths and weaknesses of the Arthritis Self-Management Program (ASMP). 2) Compare 3- and 6-week ASMP. 3) Evaluate 1.5-hour arthritis program	None	[84] Citing [85]	Patients with arthritis	USA<br>Intervention context<br>Community setting	SM	Journal article	3 studies- Study 1 : Qualitative methods incorporating incomplete block design. Study 2: Comparison of 3- and &week outcomes. Study 3: Pre-test/ post-test comparison s1N164 s2N151 s3N200
To evaluate an arthritis SM programme	None	[54]	Arthritis patients	USA<br>Intervention RCT context Community setting, includes primary care use	SM	Journal article	RCT N554
To determine the effectiveness of a SM education intervention	None	[55]	Arthritis patients with pain	USA	SM	Journal article	quantitative intervention before and

				Intervention- adult education community setting			after study, questionnaire N89
Extending current models of activity of living to include chronic pain	Coping definition	[97] Citing [77]	Chronic pain patients	Non empirical Nursing theory for practice (UK context)	Chronic pain and coping<br>Stress	Journal article	theory
Authors investigate persons with expertise in self-care management of type2 diabetes,HIV/AIDS, and multiple sclerosis	Descriptive summary of application of SM intervention	[101]	Type2 diabetes, HIV/AIDS, and multiple sclerosis	Self-care home setting	Self-care	Journal article	secondary analysis of qualitative studies, focus groups, Think aloud, interview methods (N43)
Address theory on self-care work and chronic illness	Definition	[109]	Chronically ill patients	Theory (at home setting)	Theoretical concerns of the sociology of work<br><br>Problems of managing chronic illness at home<br><br>*Three types of work and consequences of their interplay are discussed: illness work, everyday life work, and biographical work*	Book	Theory (includes case histories)

To test content validity and usability of the Organ Transplant Information System (OTISTM).	Definition	[99] Citing [25,109]	Kidney transplantation patients	Switzerland Clinical setting	self-management – including conceptual model, patient education	Journal article,	qualitative content validity of an intervention, Think Aloud method and descriptive data N 14
Short history of self-management.	Definition	[25] Citing [57,92,109]	None specified, literature review includes chronic diseases	Secondary analysis literature review	Self-Management Education:	Journal article,	qualitative content validity of an intervention, Think Aloud method and descriptive data N 14
Philosophy of meta patterns and information	None	[57]	No specific population	Theory- non empirical	Combining information meta pattern	Book,	theory
To present the Shifting Perspectives Model of Chronic Illness, which was derived from a metasynthesis of 292 qualitative research studies	None	[92]	Chronic illness	Literature synthesis Reported experience of pw chronic illness	The Shifting Perspectives Model	Journal article	meta synthesis, 292 qualitative studies N not reported
Overview of the literature (ethical analysis) of Patient adherence and patient self-management literature	Definition	[94] Citing [26,95]	SOTx patients	Overview of the literature	Patient adherence and patient self-management, ethical framework	Journal article	discussion; ethical analysis, literature overview
SM of chronic disease theory development	Definition	[95] Citing [81]	Chapters related people with chronic disease and renal transplant	Theory and literature overview<br><br>USA	SM preparation , living with chronic disease and types of work (same as Corbin and Strauss , 1988).<br>, associated learning approaches theory- social learning	journal article, review,	theory referring to literature

					theory, trans-theoretical model, belief models, problem solving		
Discussion of the rationale and definition of SM and health education SM model example application	Definition	[81]	Populations with Chronic illness	Draws on literature of primary and secondary, nursing home contexts and at home management	The SM model<br><br>SM education criteria<br><br>Acute and chronic disease differences (Holman 1992 model)	Journal article,	definitions and literature overview
To review the SM approaches of people with chronic conditions	Definition	[26] Citing [31]	Patients with chronic conditions<br>Majority of studies about asthma (66/145) includes 3 general chronic disease studies	Literature review<br>Majority of countries USA (82); UK (13) ; (10) Australia from 145	SM	Journal article	theoretical and methods overview, Literature review studies from: USA (82); 13 UK; 10 Australia N145 studies
Overview<br>of patient education interventions for people with osteoarthritis (OA) including a review of the methods used and the typical content of current educational provision for OA.	Definition	[31] Citing [61]	Osteoarthritis patients	Literature review of secondary evidence for interventions<br><br>Settings include primary care, at home, community, clinical care	Sm focus<br>Distinguish between Self-care and SM	Book (manual)	theory

Article contains tips for the home health professional on how he or she can improve the health literacy of his or her patients, which can save lives.	None	[93]	Adults with low health literacy	Management in home setting	Health literacy, Medication mismanagement	journal article	literature overview
To delineate five concepts that are often used synonymously in the nursing and related literature: self-care, self-management, self-monitoring, symptom management, and self-efficacy for self-care.	Definition	[33] Citing [34,90,98,101,102]	No specific patient focus	USA	SM	Journal article	conceptual overview
The objective of this study was to analyse and define the concept of self-management for adults diagnosed with epilepsy in the previous 12 months.	Descriptive summary of application of SM interventions	[102]	Adults newly diagnosed with epilepsy	USA<br><br>Home setting	SM	Journal article	qualitative research and literature review, semi structured interviews (N4)
Explores the development of the concept of self-care through health related literature and reviews the factors that have shaped the concept.	Definition	[34] Citing [52,64,67,87,96]	Chronic disease	Literature review	Self-care, chronic disease management; SM programmes	Journal article	literature review concept analysis
Guest Editorial: Chronic disease management and self-care support for people living with long-term conditions: is the nursing workforce prepared?	Definition	[52]	People living with long-term conditions	Secondary source review in editorial	SM<br>Self care	Journal article	Editorial
Outline of policy to meet needs of people with chronic conditions	Definition	[87]	people with chronic conditions and depression	Policy document New Zealand	SM support	Policy document	N/A

To examine ethical issues facing the SM, urging ways in which these issues must be addressed as the movement continues to evolve	Definition	[96] Citing [26]	Patients with chronic disease	Literature overview and ethical analysis	SM Self-Empowerment	Journal article	ethical analysis- literature overview
The goal of the pilot study was to evaluate the preliminary effect of an MISC intervention on HF patients' self-care behaviours.	Definition of self-care	[90]	Heart failure patients	Pilot study for an intervention (motivational interviewing via phone) recruited via tertiary care<br><br>Canada	Self-care- The Theory of HF Self-care (Rieegel et al 2008)	Journal article	pilot study randomised control group study N30
To describe a situation-specific theory of heart failure self-care in which self-care is defined as a naturalistic decision-making process involving the choice of behaviours that maintain physiologic stability (maintenance) and the response to symptoms when they occur (management)	Definition	[98] Citing [25,28,63,67,88,91]	Heart failure patients	Primary data obtained from 7 sites in the United States- setting unknown	Self-care, SM; Self care management (including a conceptual model)	Journal article	quantitative and qualitative instrument development, secondary data analysis for theory development N760
Review of literature and concepts of Self-care perspectives from six disciplines	Definition of self-care	[67] 36 Gantz, 1990	Nursing perspective	Non empirical<br><br>USA	Self-care	Journal article	Literature review
Literature overview of behavioural contributions to childhood asthma	None	[63]	Children with asthma	Literature overview	Rehabilitation, self-control	journal article	literature overview of interventions and

				Description of Children's asthma rehabilitation programme interventions (includes setting of the Centre and interventions at home)<br><br>Denver, USA			description of an intervention
This supplement to Patient reviews across the whole field of self-management of asthma by adults.	Definition	[91]	Adults with Asthma	Literature overview<br><br>Descriptions from UK	SM	Journal article-supplement<br>,	literature overview
1.Explore how patient expertise is viewed, interpreted, defined and experienced by both patients and health professionals.<br>2. Analyse how patient expertise is promoted and enabled through the self-management process.<br>3. Uncover the mechanisms that enhance or impede the development of patient expertise	Definition	[28] Citing [65,66,89,105]	Nurses' responses to expert patients with chronic conditions	A relatively affluent English county including community, primary and secondary care settings	SM	Journal article	A grounded theory approach was utilised with two concurrent data strands. Focus groups, interviews and observation., purposeful and theoretical sampling N100 health professionals (nurses, doctors, physiotherapists) and N100 adults affected by chronic disease

The aim of the study was to obtain the views and priorities of people with multiple sclerosis (MS) to inform the design of a professionally guided self-care programme	None	[88]	People with multiple sclerosis (MS)	The study was conducted at the Centre for Research in Rehabilitation at Brunel University in London, UK	Self -care	Journal article	A three-round postal Delphi survey was used as a research to study N136
Review, on what is meant by the term ''asthma self-management'', how self-management plans are developed and implemented, the evidence for their efficacy, what models are available, what patients seem to want, which asthmatics need to follow self-manage-compliance with this system of care.	Definition	[65]	Asthma patients	Literature review , international	SM Models: Asthma self-management plan promoted by National Asthma Campaign (UK). Booklet 7: 'Self-management and peak flow measurement'.	Journal article	literature overview
Reports on a pilot study on the effects of self-management in COPD suggest a reduction of morbidity due to COPD.	Definition	[105] .	COPD patients	Out-patient setting Germany	SM behaviours, educational programmes	journal article	Before and after trial of a an intervention, N19
This paper aims to provide guidance for clinicians, based on the evidence available regarding the clinical effectiveness and health economics of the self management of oral anticoagulation therapy by patients	Definition	[66]	Patients undertaking self-management of oral anticoagulation.	Review of literature<br><br>UK perspective, international evidence	SM, near patient device (CoaguChek, Roche Diagnostics, Mannheim), health technology assessment	journal article	Literature overview

Behavioural study to assess whether high generalised and asthma-specific self-efficacy expectancies were predictive of adequate self-management and self-treatment behaviour.	Descriptive summary of application of SM intervention	[89] Citing [53]	Patient with asthma and COPD	Population survey , home setting self-treatment<br><br>Netherlands	SM,<br>Social support, self-efficacy; social-learning theory of Bandura	journal article,	Behavioural study Questionnaire A N4563 persons (18–65 years) who had used inhaled medication in 1993- of which N1262 were asthmatic patients
To present theory	Definition of self-efficacy	[53]	SM of pain relates to heart attack patients example	SM example is based in context of pain cognitive appraisal in everyday life<br><br>USA	SM – only in relation n to coping with pain , including psychosocial influencers<br>Social cognitive/learning theory. Theory of human nature and causality, (within self-efficacy theory concepts relate to generative capability, competent functioning , faulty assessment, perceived self-efficacy , health behaviour	Book	theory
