# Supplementary material for: Defining Self-Management for Solid Organ Transplantation Recipients: A Mixed Method Study
Source: Nurs Rep. 2024 Apr 17;14(2):961–87. doi: 10.3390/nursrep14020073 (PMC11036239; doi:10.3390/nursrep14020073)
Supplement: Supplementary file 1 [file nursrep-14-00073-s001.zip › S4.pdf]

Supplementary File S4 (S4): Additional information about the expert feedback summary

First iteration of definition from cluster headings:

Self-management (SM) for Solid Organ Transplant recipients is a multi-step and iterative process taking place over the lifetime and therefore conceptually linked to living indefinitely with chronic illness. This process is undertaken in order to optimise transplant outcomes and to live well, occurring in conjunction with loved ones and health professionals who may act as external support to promote SM. Practically, SM concerns different activities and tasks in three types of work (i.e. managing emotions, everyday life, and medical regimen) and requires specific competencies (knowledge, skills, and attitudes). Active engagement in healthy behaviours is important. Patient prioritisation of tasks and decision-making facilitated by traits of control, structure and discipline are central characteristics; as are the moderating factors of patient motivation, self-efficacy, and cognitive function.

Experts noted aspects we agreed with and amended:

1. Feedback prompted the team to consider if a description of the SM process as iterative was inconsistent with Bandura's 1977 Self Efficacy Theory. The principal assumptions are that, psychological treatments can initiate and improve self-efficacy (and strengthen expectations of personal efficacy) to help people perform better on behavioural tasks. We identified self-efficacy as a moderating factor as opposed to a prerequisite behavioural component. Although, this should be tested in further research on the proposed definition.
2. The phrasing of active engagement was contested with reference to Lorig's Theory: One „cannot not manage" Lorig & Holman, 2003, p.1- ". After discussion we considered active engagement an appropriate term as it is possible for patients to manage poorly and not to engage in SM. However, we acknowledge also the same concept is echoed in our data by Schafer-Keller et al 2009 "Whether one is engaging in a health promoting activity such as exercise or is living with a chronic disease such as asthma, he or she is responsible for day-to-day management…Unless one is totally ignorant of healthful behaviours it is impossible not to manage one's health."
3. Experts noted the concept of living well lacks objective measurement in (however there may be other associated scales)
4. Active engagement in healthy behaviours is important – remove this indicator of significance from definition
5. The team considered if motivation was dichotomous- we conceptualised it as on a continuum
6. We were prompted to consider levels of similarity in the definition between SOTx and chronically ill patients SM – we therefore identified specific components for SOTx patients

Experts raised points which we were unable to find evidence to support in the data, despite our support:

1. The paternalistic phrasing i.e. SM should be associated with the patient and not the transplant.
2. Inclusion of peers as a form of informal support
3. Societal level contextualisation for SM- we acknowledge this aspect as a limitation of the data.
